# Supplementary material for: Acceptability and perceived barriers to reactive focal mass drug administration in the context of a malaria elimination program in Magude district, Southern Mozambique: A qualitative study
Source: PLoS One. 2023 Mar 31;18(3):e0283160. doi: 10.1371/journal.pone.0283160 (PMC10065238; doi:10.1371/journal.pone.0283160)
Supplement: S1 Appendix — (DOCX) [file pone.0283160.s001.docx]

**S1A Appendix. Semi-structured interview (SSI) guide for household heads, women of reproductive age, adolescents, members of the general community and community leaders (Portuguese version)**

1. **DADOS DO ENTREVISTADO**

| **Ref./Ficheiro/Audio**  **(REACT-SOC-ESE-*NumESE-mmdd*)** | REACT-SOC-ESE-\|__\|__\|-\|__\|__\|__\|__\| |
| --- | --- |
| **Data** | \|__\|__\|-\|__\|__\|-\|__\|__\|__\|__\| |
| **Local Específico** | \|__\| Especificar: ______________________________ |
| **Género do participante** | □ Masculino □ Feminino |
| **Situação Marital** | □ Solteiro/a □ Casado/a □União□Viúvo/a □NA□Outro (especificar) ______________________ |
| **Nível de Escolaridade** | □ Nenhum □ Primária □ Secundária □ Superior |
| **Ocupação** | □ Doméstico □ Camponês □ Camponês remunerado □ Estudante □ Negociante □ Serviços  □ Trabalhador da Saúde □ Outro (especificar): ­____________________________________________ |
| **Religião** | □Cristão □Islâmica □ Hindu □ Animista □ Ateus □ Outro(especificar): _______________________________ |
| **Hora de Início da ESE** | \|__\|__\|:\|__\|__\| |
| **Hora do Fim da ESE** | \|__\|__\|:\|__\|__\| |
| **Resultado da ESE** | □ Completa □Incompleta, razões: ________________________________________________  Se aplicável, remarcada para: \|__\|__\|-\|__\|__\|-\|__\|__\|__\|__\| |

1. **CONTEÚDOS DA ENTREVISTA**

| 1. **Conhecimentos sobre a malária** 2. Na sua opinião, quando uma pessoa tem febres, arrepios de frio, dores de cabeça e as vezes vómitos, que doença deve ter?  - Caso refira outra doença, perguntar: - Se já ouviu falar de malária? - Quais são os sintomas?  1. O que sabe mais sobre a malaria?  - Causa - Prevenção/ Explorar mais sobre a prevenção - Tratamento  1. **Percepções sobre as actividades de eliminação de malária no distrito de Magude**  - Explicar de forma geral que houve em Magude uma campanha de administração de fármaco anti-malárico.  1. Participou nas actividades de administração massiva de tratamento anti-malárico que tiveram lugar no ano de 2016 e em Janeiro de 2017 no distrito de Magude? Se não porquê?  - Explorar em detalhe o que é que aconteceu segundo o entrevistado (procedimentos, percepção dos principais intervenientes – que organizações estavam envolvidas) - Procurar saber o termo que ele(a) usa para se referir a esta campanha.  1. O que se pretendia com a campanha?  - Acha que estes objectivos foram atingidos? - Em que medida? De que forma?  1. Que impacto acha que estas actividades tiveram ou estão a ter na comunidade?  - Aspectos positivos - Aspectos negativos - Se o participante menciona que “a malária baixou”, como é que ele nota que a malária baixou?  1. Acha que a malária ainda é um problema de saúde na comunidade?  - Se o participante disser que sim, explorar se mudou o comportamento em relação à prevenção de malária (uma vez que ele considera que a malária já não é um problema).  1. **Aceitabilidade** **da investigação de casos de Malária reactiva** 2. Já ouviu falar das investigações de casos de malária que estão a acontecer agora na comunidade? (falar particularmente sobre os MDAs focais como reacção a um caso de malária que apareceu na Unidade Sanitária).  - Descrever a origem da informação (canais de comunicação natureza dos informantes) - Quais são as fontes de informação mais credíveis para vocês?   - Porquê? Porquê não? - Participou nestas actividades? Se sim porquê? Se não porquê? - Como se sentiu por ter participado nesta actividade? - Conhece alguma pessoa/família que participou? - O que se pretendia com essa actividade? - Acha que os fMDAs serão importantes para a comunidade? Se sim /porquê? Se não porquê?  1. Acha que a comunidade aceita este tipo de intervenção, de testarem alguém na Unidade Sanitária, caso o teste de Malária for positivo, irem testar pessoas da sua casa e os seus vizinhos para saber se terão o parasita ou não e se tiver tratarem na hora? 2. Se algum membro do seu agregado ou seu vizinho for identificado como caso de malária, você estaria disposto a fazer o teste de malária e tomar a medicação mesmo se o resultado do teste for negativo? 3. Quais são ou serão os problemas e barreiras que a equipa poderá encontrar na comunidade na hora de pôr em prática estas actividades? 4. Acha que é importante eliminarmos a malária em Moçambique? |
| --- |
| 1. **(SÓ PARA AS MULHERES EM IDADE REPRODUCTIVA): Percepções em relação aos procedimentos nas mulheres em idade reprodutiva e as mulheres grávidas.** 2. O que é que você acha em relação a testagem de gravidez a todas as mulheres em idade reproductiva, na hora de irem fazer o teste de malária á todos membros do agregado e os vizinhos? 3. Saberia dizer qual foi a razão pela qual as mulheres em idade reprodutiva terão de fazer o teste de gravidez? 4. Que pessoas da comunidade em especial acham que podem não gostar da testagem de gravidez, entre as mulheres em idade reprodutiva? 5. **Motivações e barreiras para participação e aderência ao tratamento da malária** 6. Quais são as principais razões pelas quais você e as pessoas da comunidade aceitariam participar nestas actividades de eliminação de malária? 7. O que é que acha em relação ao medicamento anti-malárico que está a ser administrado? 8. Quais serão as barreiras que a equipe poderá encontrar na comunidade, na hora de pôr em prática as suas actividades? 9. **Recomendações para a melhoria das actividades de eliminação de malária no futuro e para melhorar aderência**    - - 1. Que recomendações tem a deixar para melhorar a toma de medicamentos, aceitabilidade do teste de gravidez e da malaria? |
|  |

1. **OBSERVAÇÕES**

NOME DO ENTREVISTADOR: ______________________________ Assinatura: _______________________ CÓDIGO: |__|__|__|__
