# Supplementary material for: Acceptability and perceived barriers to reactive focal mass drug administration in the context of a malaria elimination program in Magude district, Southern Mozambique: A qualitative study
Source: PLoS One. 2023 Mar 31;18(3):e0283160. doi: 10.1371/journal.pone.0283160 (PMC10065238; doi:10.1371/journal.pone.0283160)
Supplement: S2 Appendix — (DOCX) [file pone.0283160.s002.docx]

**S1B Appendix. Semi-structured interview (SSI) guide for household heads, women of reproductive age, adolescents, members of the general community and community leaders (English Version)**

1. INTERVIEWEE DATA

| **Ref./File/Audio**  **(REACT-SOC-ESE-*NumESE-mmdd*)** | REACT-SOC-ESE-\|__\|__\|-\|__\|__\|__\|__\| |
| --- | --- |
| **Date** | \|__\|__\|-\|__\|__\|-\|__\|__\|__\|__\| |
| **Specific place** | \|__\| Specify: ______________________________ |
| **Participant initials** | \|__\|__\| |
| **Gender** | □ Male □ Female |
| **Marital status** | □ Single □ Married □Union □Widow □NA □Other (specify) ______________________ |
| **Level of education** | □ None □ Primary □ Secondary □ High education |
| **Occupation** | □ Housemaid □ Famer □ Paid farmer □ Student □ salesman/saleswoman □ Service  □ Health worker □ Other (specify): ­____________________________________________ |
| **Religion** | □Cristian □Muslim □ Hindu □ Animist □ Atheist □ Other(specify): _______________________________ |
| **Starting time of the interview** | \|__\|__\|:\|__\|__\| |
| **Ending time of the interview** | \|__\|__\|:\|__\|__\| |
| **Result of the semi-structured interview** | □ Complete □Incomplete, reasons: ________________________________________________  IF applicable, remarkable for: \|__\|__\|-\|__\|__\|-\|__\|__\|__\|__\| |

**II. Content of the interview**

| **I. Malaria knowledge**  1. In your opinion, when a person has fevers, chills, headache and sometimes vomiting, which disease should they have?   - If she/he mentions another disease, ask: - If she/he has heard of malaria? - What are the symptoms?   2. What else do you know about malaria?   - Cause - Prevention/Exploring more about prevention - Treatment   **II. Perceptions on malaria elimination activities in Magude district**   - Explain generally that there was a campaign of administration of anti-malarial drug in Magude.   1. Did you participate in the mass anti-malarial treatment administration activities that took place in the year 2016 and January 2017 in Magude district? If not why?   - Explore in detail what happened according to the interviewee (procedures, perception of key players - what organisations were involved). - Seek to know the term he/she uses to refer to this campaign.   2. What was the aim of the campaign?   - Do you think these objectives were achieved? - To what extent? In what way?   3. What impact do you think these activities had or are having in the community?   - Positive aspects - Negative aspects - If the participants mention that "malaria has gone down", how do they note that malaria has gone down?   4. Do you think malaria is still a health problem in the community?   - If the participants say: yes, explore whether they have changed their behaviour regarding malaria prevention (once they consider malaria no longer a problem).   **III. Acceptability of Reactive Malaria case investigations 1.**  1. Have you heard about the malaria case investigations that are now happening in the community? (talk particularly about focal MDAs as a reaction to a malaria case that appeared in the Health Unit).   - Describe the source of information (channels of communication nature of informants) - What are the most credible sources of information to you?   - Why? Why not? - Did you participate in these activities? If yes why? If no, why not? - How did it feel to have participated in this activity? - Do you know any person/family who participated? - What was the purpose of this activity? - Do you think the fMDAs will be important to the community? If yes/why? If no, why not?   2. Do you think that the community accepts this type of intervention, to test someone in the Health Unit, if the Malaria test is positive, to go and test people from their house and their neighbours to know if they have the parasite or not and if they do have it, to treat it on the spot?  3. If any member of your household or your neighbour is identified as having malaria would you be willing to take a malaria test and take medication even if the test result is negative?  4. What are or will be the problems and barriers that the team may encounter in the community when implementing these activities?  5. Do you think it is important that we eliminate malaria in Mozambique?  **IV. (ONLY FOR WOMEN OF REPRODUCTIVE AGE): Perceptions regarding procedures in women of reproductive age and pregnant women.**  1. What do you think about pregnancy testing for all women of reproductive age, going to test all household members and neighbours for malaria?  2. Can you tell us the reason why women of reproductive age will have to do the pregnancy test?  3. Which people in the community in particular do you think might not like pregnancy testing among women of reproductive age?  **V. Motivations and barriers to participation and adherence in malaria treatment**  1. What are the main reasons why you and people in the community would agree to participate in these malaria elimination activities?  2. How do you feel about the anti-malarial drug being administered?  3. What barriers might the team encounter in the community when implementing their activities?  **VI. Recommendations for improving future malaria elimination activities and improving adherence**  What recommendations do you have for improving medication uptake, acceptability of pregnancy test and malaria test? |
| --- |

1. REMARKS

NAME OF INTERVIEWER: ______________________________ Signature: _______________________ CODE: |__|__|__|
