# Supplementary material for: Acceptability and perceived barriers to reactive focal mass drug administration in the context of a malaria elimination program in Magude district, Southern Mozambique: A qualitative study
Source: PLoS One. 2023 Mar 31;18(3):e0283160. doi: 10.1371/journal.pone.0283160 (PMC10065238; doi:10.1371/journal.pone.0283160)
Supplement: S3 Appendix — (DOCX) [file pone.0283160.s003.docx]

**S2A Appendix. Semi-structured interview (SSI) guide for healthcare professionals and community health workers (Portuguese version)**

1. **DADOS DO ENTREVISTADO**

| **Ref./Ficheiro/Audio**  **(REACT-SOC-ESE-*NumESE-mmdd*)** | REACT-SOC-ESE-\|__\|__\|-\|__\|__\|__\|__\| |
| --- | --- |
| **Data** | \|__\|__\|-\|__\|__\|-\|__\|__\|__\|__\| |
| **Local Específico** | \|__\| Especificar: ______________________ |
| **Género do participante** | □ Masculino □ Feminino |
| **Situação Marital** | □ Solteiro/a □ Casado/a □União□Viúvo/a □NA□Outro (esp) ______________________ |
| **Nível de Escolaridade** | □ Nenhum □ Primária □ Secundária □ Superior |
| **Ocupação** | □ Trabalhador da Saúde □ Especifique a função: ­_________________________________ |
| **Religião** | □Cristão □Islâmica □ Hindu □ Animista □ Ateus □ Outro(esp): _______________________________ |
| **Hora de Início da ESE** | \|__\|__\|:\|__\|__\| |
| **Hora do Fim da ESE** | \|__\|__\|:\|__\|__\| |
| **Resultado da ESE** | □ Completa □Incompleta, razões: ________________________________________________  Se aplicável, remarcada para: \|__\|__\|-\|__\|__\|-\|__\|__\|__\|__\| |

1. **CONTEÚDOS DA ENTREVISTA**

| 1. **Percepções sobre as actividades de eliminação de malária no distrito de Magude**  - Há quanto tempo está no seu actual posto? - Tomou o medicamento durante as actividades de administração massiva de tratamento antimalárico que tiveram lugar no ano de 2016 no distrito de Magude? Se não porquê? - Teve alguma função na implementação das actividades?   - Se sim, especificar - Explorar em detalhe o que é que aconteceu segundo o entrevistado (procedimentos, percepção dos principais intervenientes – que organizações estavam envolvidas) - O que se pretendia com a campanha? - Acha que estes objectivos foram atingidos? - Na sua opinião, que instituições estão a liderar estas actividades na prática? - Que impacto acha que estas actividades tiveram ou estão a ter nas unidades sanitárias? - Aspectos positivos - Aspectos negativos - Se o participante menciona que “a malária baixou”, como é que ele nota que a malária baixou - Acha que é prioritário eliminarmos a malária na comunidade?/ Se sim, porquê?/ Se não porquê? - Acha que a malária é problema na comunidade?   - Se o participante disser que sim, explorar se o comportamento dos seus colegas em relação ao diagnostico de malária (uma vez que ele considera que a malária já não é um problema) |
| --- |

1. **OBSERVAÇÕES _________________________________________________________________**

NOME DO ENTREVISTADOR: ________________________ Assinatura: _______________ CÓDIGO: |__|__|__|__|
