# Supplementary material for: Acceptability and perceived barriers to reactive focal mass drug administration in the context of a malaria elimination program in Magude district, Southern Mozambique: A qualitative study
Source: PLoS One. 2023 Mar 31;18(3):e0283160. doi: 10.1371/journal.pone.0283160 (PMC10065238; doi:10.1371/journal.pone.0283160)
Supplement: S4 Appendix — (DOCX) [file pone.0283160.s004.docx]

**S2B Appendix. Semi-structured interview (SSI) guide for healthcare professionals and community health workers (English Version)**

| **Ref./File/Audio**  **(REACT-SOC-ESE-*NumESE-mmdd*)** | REACT-SOC-ESE-\|__\|__\|-\|__\|__\|__\|__\| |
| --- | --- |
| **Date** | \|__\|__\|-\|__\|__\|-\|__\|__\|__\|__\| |
| **Specific place** | \|__\| Specify: ______________________________ |
| **Participant initials** | \|__\|__\| |
| **Gender** | □ Male □ Female |
| **Marital status** | □ Single □ Married □Union □Widow □NA □Other (specify) ______________________ |
| **Level of education** | □ None □ Primary □ Secondary □ High education |
| **Occupation** | □ Health worker □ Other (specify): ­____________________________________________ |
| **Religion** | □Cristian □Muslim □ Hindu □ Animist □ Atheist □ Other(specify): _______________________________ |
| **Starting time of the interview** | \|__\|__\|:\|__\|__\| |
| **Ending time of the interview** | \|__\|__\|:\|__\|__\| |
| **Result of the semi-structured interview** | □ Complete □Incomplete, reasons: ________________________________________________  Not applicable, remarkable for: \|__\|__\|-\|__\|__\|-\|__\|__\|__\|__\| |

| **II. Content of the interview**  I. **Perceptions of malaria elimination activities in Magude district**   - How long have you been in your current post? - Did you take the medicine during the mass antimalarial treatment administration activities that took place in the year 2016 in Magude district? If not why? - Did you have any role in the implementation of the activities? - If yes, please specify. - Explore in detail what happened according to the interviewee (procedures, perception of key players - which organisations were involved). - What was the aim of the campaign? - Do you think these objectives were achieved? - In your opinion, which institutions are leading these activities in practice? - What impact do you think these activities had or are having on the health facilities? - Positive aspects - Negative aspects - If the participant mentions that "malaria has gone down", how do you perceive that malaria has gone down? - Do you think it is a priority to eliminate malaria in the community? If yes, why? If no, why not? - Do you think malaria is a problem in the community? - If the participant says yes, explore their colleagues' behaviour in relation to malaria diagnosis (as they feel malaria is no longer a problem). |
| --- |

1. Remarks

NAME OF INTERVIEWER: ______________________________ Signature: _______________________ CODE: |__|__|__|
