# Supplementary material for: Acceptability and perceived barriers to reactive focal mass drug administration in the context of a malaria elimination program in Magude district, Southern Mozambique: A qualitative study
Source: PLoS One. 2023 Mar 31;18(3):e0283160. doi: 10.1371/journal.pone.0283160 (PMC10065238; doi:10.1371/journal.pone.0283160)
Supplement: S5 Appendix — (DOCX) [file pone.0283160.s005.docx]

**S3A Appendix. Focus groups discussion (FGD) guide for general population: men and women (Portuguese version)**

| Local onde DGF ocorreu: \|__\|__\| DGF Número: \|__\|__\| Data: \|__\|__\|-\|__\|__\|-\|__\|__\|__\|__\| Facilitador: \|__\|__\|__\| Redactor_ \|__\|__\|__\| |
| --- |

1. **INFORMAÇÃO DEMOGRÁFICA DOS PARTICIPANTES**

| **Part.** | **Idade** | **Sexo**  **(F/M)** | **Situação Marital (*1)** | **Nível**  **Escolaridade (*2)** | **Ocupação**  **(*3)** | **Religião**  **(*4)** | **Posto**  **Administrativo (*5)** |
| --- | --- | --- | --- | --- | --- | --- | --- |
| 1 | \|__\|__\| | \|__\| |  | \|__\| |  |  | \|__\| |
| 2 | \|__\|__\| | \|__\| |  | \|__\| |  |  | \|__\| |
| 3 | \|__\|__\| | \|__\| |  | \|__\| |  |  | \|__\| |
| 4 | \|__\|__\| | \|__\| |  | \|__\| |  |  | \|__\| |
| 5 | \|__\|__\| | \|__\| |  | \|__\| |  |  | \|__\| |
| 6 | \|__\|__\| | \|__\| |  | \|__\| |  |  | \|__\| |
| 7 | \|__\|__\| | \|__\| |  | \|__\| |  |  | \|__\| |
| 8 | \|__\|__\| | \|__\| |  | \|__\| |  |  | \|__\| |
| 9 | \|__\|__\| | \|__\| |  | \|__\| |  |  | \|__\| |
| 10 | \|__\|__\| | \|__\| |  | \|__\| |  |  | \|__\| |
| 11 | \|__\|__\| | \|__\| |  | \|__\| |  |  | \|__\| |
| 12 | \|__\|__\| | \|__\| |  | \|__\| |  |  | \|__\| |
| ***1. Situação Marital**: 1-Solteiro/a 2-Casado/a 3-União 4-Viúvo/a 5-Outro (especificar)  ***2. Nível de Escolaridade**: 1-Nenhum 2-Primária 3-Secundária 4-Superior  ***3. Ocupação**: 1- Doméstico 2- Camponês 3- Camponês remunerado 4- Estudante 5-Negociante 6-Serviços 7-Trabalhador da Saúde 8-Outro (esp)  ***4. Religião:** 1-Cristão 2-Islâmico 3-Hindu 4-Animista 5-Ateus 9-Outro(esp)  ***5. Posto Administrativo:** 1-Magude Sede 2-Motaze 3-Panjane 4-Mahele 5-Mapulanguene | | | | | | | |

1. **DADOS DA DGF**

| **Ref. DGF/Ficheiro/Áudio**  **(REACT-SOC-DGF-*NumDGF-mmdd*)** | REACT-SOC-DGF-\|__\|__\|-\|__\|__\|__\|__\| |
| --- | --- |
| **Data** | \|__\|__\|-\|__\|__\|-\|__\|__\|__\|__\| |
| **Local Específico (ex: círculo, escola)** |  |
| **Número Inicial de Participantes** | \|__\|__\| |
| **Número Final de Participantes** | \|__\|__\| |
| **Hora de Inicio da DGF** | \|__\|__\|:\|__\|__\| |
| **Hora do Fim da DGF** | \|__\|__\|:\|__\|__\| |
| **Resultado da DGF** | \|__\| Completa \|__\| Incompleta, razões:  ________________________________________________  Se aplicável, remarcada para: \|__\|__\|-\|__\|__\|-\|__\|__\|__\|__\| |

1. **CONTEÚDOS DA DISCUSSÃO**

| 1. **Conhecimento sobre malária e conceito de “eliminação”** 2. Na vossa opinião, quando uma pessoa tem febres, arrepios de frio, dores de cabeça e as vezes vómitos, que doença pode ter? 3. O que sabem mais sobre esta doença?  - Causa - Prevenção/ Explorar mais sobre a prevenção - Tratamento - Será que sempre que uma pessoa tem febre, vai a Unidade Sanitária?   - Quais são os outros provedores que procuram?  1. Caso não tenham usado o termo malária, procurar saber se a doença que descreveram têm há ver com malária ou não?  - Se não, debater as diferenças, e procurar saber sobre causas, prevenção, tratamento da malária  1. Na vossa opinião, acham que é possível eliminar (acabar com a malária) no vosso distrito?  - Descrever argumentos a favor e contra esta ideia - Explorar os termos localmente usados para o conceito “eliminação” e usá-los sempre que possível      1. Já ouviram falar da campanha de eliminação de malária que houve no distrito de Magude no ano de 2016 e em Janeiro de 2017?  - Participaram nesta campanha? Se sim, porquê? /Se não porquê? - O que se pretendia com essa Campanha? - Acham que estes objectivos foram atingidos?  1. Que impactos acham que estas actividades tiveram ou estão a ter nas unidades sanitárias?  - Aspectos positivos - Aspectos negativos  1. **Aceitabilidade da comunidade em relação** **a investigação de casos de Malária** 2. Já ouviram falar das investigações de casos de malária que estão a acontecer agora na comunidade? (a falar particularmente sobre os MDAs focais como reacção a um caso de malária que apareceu na unidade sanitária)  - Descrever a origem da informação (canais de comunicação natureza dos informantes) - Quais são as fontes de informação mais credíveis para vocês?   - Porquê? Porquê não? - Participaram nestas actividades? Se sim, porquê? /Se não porquê? - Conhecem alguma pessoa/família que participou? - O que se pretendia com essa actividade? - Acham que os fMDAs serão importantes para a comunidade? Se sim /porquê? Se não porquê? - **Se nenhum participante ouviu falar das actividades, explicar.**  1. O que é que vocês acham destas Actividades de um modo geral?  - Descrever as suas opiniões e discussão  1. Acham que a comunidade aceita esta intervenção?  - Discutir as razões da aceitabilidade e não aceitabilidade - Que grupos seriam os mais resistentes, porquê? - Que grupos seriam mais colaboradores, porquê?  1. Quais esperam que sejam os desafios e barreiras que a equipa de estudo irá encontrar na comunidade, na hora de pôr em prática as actividades de investigação de casos na comunidade?  - Testes rápidos de malária - Testes de gravidez - Administração do medicamento.   **III – Recomendações do grupo para maximização do sucesso das actividades**  **Discussão livre**:   - Orientar aos participantes no sentido de falarem abertamente sobre o que recomendariam as equipes de mobilização; as equipes de implementação e a comunidade em geral. |
| --- |

1. **OBSERVAÇÕES**

**ASSINATURAS**

NOME DO FACILITADOR: _________________________________ Assinatura: _______________________ CÓDIGO: |__|__|__|__|

NOME DO REDACTOR: ____________________________________ Assinatura: _______________________ CÓDIGO: |__|__|__|__|
