# Supplementary material for: Acceptability and perceived barriers to reactive focal mass drug administration in the context of a malaria elimination program in Magude district, Southern Mozambique: A qualitative study
Source: PLoS One. 2023 Mar 31;18(3):e0283160. doi: 10.1371/journal.pone.0283160 (PMC10065238; doi:10.1371/journal.pone.0283160)
Supplement: S6 Appendix — (DOCX) [file pone.0283160.s006.docx]

**S3B Appendix. Focus groups discussion (FGD) guide for general population: men and women (English version)**

Local code: |__|__| FGD Number: |__|__| Date: |__|__|-|__|__|-|__|__|__|__| Facilitator code: |__|__|__| Redactor_ |__|__|__|

1. **Demographic information of the participants**

| **Participant** | **Age** | **Sex**  **(F/M)** | **Marital status (*1)** | **Level of education (*2)** | **Occupation**  **(*3)** | **Religion**  **(*4)** | **Administrative Post (*5)** |
| --- | --- | --- | --- | --- | --- | --- | --- |
| 1 | \|__\|__\| | \|__\| |  | \|__\| |  |  | \|__\| |
| 2 | \|__\|__\| | \|__\| |  | \|__\| |  |  | \|__\| |
| 3 | \|__\|__\| | \|__\| |  | \|__\| |  |  | \|__\| |
| 4 | \|__\|__\| | \|__\| |  | \|__\| |  |  | \|__\| |
| 5 | \|__\|__\| | \|__\| |  | \|__\| |  |  | \|__\| |
| 6 | \|__\|__\| | \|__\| |  | \|__\| |  |  | \|__\| |
| 7 | \|__\|__\| | \|__\| |  | \|__\| |  |  | \|__\| |
| 8 | \|__\|__\| | \|__\| |  | \|__\| |  |  | \|__\| |
| 9 | \|__\|__\| | \|__\| |  | \|__\| |  |  | \|__\| |
| 10 | \|__\|__\| | \|__\| |  | \|__\| |  |  | \|__\| |
| 11 | \|__\|__\| | \|__\| |  | \|__\| |  |  | \|__\| |
| 12 | \|__\|__\| | \|__\| |  | \|__\| |  |  | \|__\| |
| ***1. Marital Status**: 1-Single 2-Married 3-Union 4-Widow 5-Other (specify)  ***2. Level of education**: 1-Nenhum 2-Primária 3-Secundária 4-Superior  ***3. Occupation**: 1- Housemaid 2- Farmer 3- Paid farmer 4- Student 5-saleman/woman 6-Service 7-Health worker 8-Other (specify)  ***4. Religion:** 1-Cristian 2-Islum 3-Hindu 4-Animist 5-Atheist 9-Other (specify)  ***5. Administrative Post:** 1-Magude village 2-Motaze 3-Panjane 4-Mahele 5-Mapulanguene | | | | | | | |

1. **FGD DATA**

| **Ref. DGF/File/Audio**  **(REACT-SOC-DGF-*NumDGF-mmdd*)** | REACT-SOC-DGF-\|__\|__\|-\|__\|__\|__\|__\| |
| --- | --- |
| **Data** | \|__\|__\|-\|__\|__\|-\|__\|__\|__\|__\| |
| **Specific local where the FGD took place** |  |
| **Number of the participants** | \|__\|__\| |
| **Beginning time** | \|__\|__\|:\|__\|__\| |
| **Ending time** | \|__\|__\|:\|__\|__\| |
| **FGD result** | \|__\| Complete \|__\| Incomplete, reasons:  ________________________________________________  If applicable, remarked for: \|__\|__\|-\|__\|__\|-\|__\|__\|__\|__\| |

1. **Content of the discussion**

| **I. Knowledge on malaria and the concept of "elimination**  3. In your opinion, when a person has fevers, chills, headache and sometimes vomiting, what illness can he or she has?  4. What do you know more about this disease?   - Cause - Prevention/ Exploring more about prevention - Treatment - Does every time a person has a fever goes to the Health Unit?   - What are the other providers people seek?   5. If they did not use the term malaria, ask if the illness they described is related to malaria or not?   - If not, discuss the differences, and find out about causes, prevention, treatment of malaria.   6. In your opinion, do you think it is possible to eliminate (stop malaria) in your district?   - Describe arguments for and against this idea. - Explore locally used terms for the concept "elimination" and use them where possible.     7. Have you heard about the malaria elimination campaign that took place in Magude district in the year 2016 and in January 2017?   - Did you participate in this campaign? If yes, why? /If no why? - What was intended with this Campaign? - Do you think these objectives were achieved?   8. What impact do you think these activities had or are having on the health units?   - Positive aspects - Negative aspects   **II. Community acceptability of Malaria case investigations**  6. Have you ever heard about the malaria case investigations that are now happening in the community? (particularly talking about focal MDAs as a reaction to a case of malaria that has appeared in the health facility).   - Describe the source of information (channels of communication nature of informants) - What are the most credible sources of information to you?   - Why? Why not? - Did you participate in these activities? If yes, why? If no why? - Do you know any person/family who participated? - What was the purpose of the activity? - Do you think the fMDAs will be important for the community? If yes /why? If no, why not? - If no participants have heard of the activities, explain.   7. What do you think about these Activities in general?   - Describe your opinions and discussion.   8. Do you think the community accepts this intervention?   - Discuss the reasons for acceptability and non-acceptability - Which groups would be most resistant, why? - Which groups would be most supportive, why?   9. What do you expect to be the challenges and barriers that the study team will encounter in the community when implementing case-finding activities in the community?   - Rapid malaria tests - Pregnancy tests - Drug administration.   **III - Recommendations from the group for maximizing the success of the activities.**  **Free discussion:**  Direct participants to talk openly about what they would recommend the mobilization teams; the implementation teams and the community at large. |
| --- |

1. Remarks: __________________________________________________________________

SIGNATURES:

FACILITATOR'S NAME: _________________________________ Signature: _______________________ CODE: |__|__|__|

NAME OF EDITOR: ____________________________________ Signature: _______________________ CODE: |__|__|_|
