# Supplementary material for: Acceptability and perceived barriers to reactive focal mass drug administration in the context of a malaria elimination program in Magude district, Southern Mozambique: A qualitative study
Source: PLoS One. 2023 Mar 31;18(3):e0283160. doi: 10.1371/journal.pone.0283160 (PMC10065238; doi:10.1371/journal.pone.0283160)
Supplement: S1 Table — (DOCX) [file pone.0283160.s007.docx]

**S4 Table. Consolidated criteria for reporting qualitative studies (COREQ): 32-item checklist**

| **No** | **Item** | **Guide question/description** | **Answer** |
| --- | --- | --- | --- |
|  | Domain 1: Research team and Reflexivity |  | |
|  | Personal characteristics |  |  |
| 1 | Interview/facilitators | Which author/s coordinated the interview or focus groups? | Helder Djive, Hoticha Nhantumbo and Ilda Murato |
| 2 | Credentials | What were the researcher's credentials? | Graduation (Bsc degree) |
| 3 | Occupation | What was their occupation at the time of the study | All were Junior Researchers |
| 4 | Gender | Was the researcher male or female? | 2 Female and 1 Male |
| 5 | Experience and training | What experience and training did the researcher have? | Qualitative analysis, ICH Good Clinical Practices, Training in qualitative data collection tools, Training in Qualitative Data Analysis using Nvivo 11, Training in Implementation Science, Gender training, and REDcap Training for Data Management |
|  | Relationship with participants | | |
| 6 | Relationship established | Was a relationship established prior to study the commencement? | Yes. |
| 7 | Participant knowledge of the interviewer | What did participants know about the researcher | Reasons for doing a research and the institution origin of the researchers. |
| 8 | Interviewer characteristics | What characteristics were reported about the interviewer/facilitator? | Bias regarding participant selection. |
|  | Domain two: Study design | | |
| 9 | Methodological orientation and theory | What methodological orientation was stated to underpin the study? | Qualitative methodology, particularly qualitative generic study |
|  | Participant Selection | | |
| 10 | Sampling | How were participants selected? | Purposive sampling |
| 11 | Methodology of approach | How were participants approached? | Face-to-face |
| 12 | Sample size | How many participants were in the study? | 239 participants considered eligible for data analysis: 69 participants from different community groups participated in semi-structured interviews; 157 participants (general population of the community) participates in focus group discussion, 9 healthcare professionals and 4 community health workers. |
| 13 | No-participation | How many people refused to participate or dropped out? Reasons | None. |
|  | Setting | | |
| 14 | Setting of data collection | Where was the data collected? | Semi-structured interviews took place in the household of the participants, focus group with the general population were conducted in the community, in a place chosen by the participants, semi-structured interviews with healthcare professionals took place in their working place – healthcare facility, and semi-interviews with community health workers were conducted in their working places – health post. |
| 15 | Presence of non-participants | Was anyone else present beside the participant and the researcher? | No. |
| 16 | Description of sampling | What are the important characteristics of the sampling? | Sex, education and occupation. |
|  | Data collection | | |
| 17 | Interview guide | Where questions, prompts, guides provided by the authors? Was it pilot tested? | Yes, the semi-structured guide and focus group discussion guide were designed and pilot tested to participants with similar characteristics of the study group in a different setting of the main study setting. The final guide was revised, and approved by all authors. |
| 18 | Repeated interviews | Were repeated interviews carried out? | No. But some participants were involved in both semi-structured interviews and focus group discussion, as some participants had multiple categories. |
| 19 | Audio/visual recording | Did the researcher use audio or visual recording to collect the data? | Yes, audio recording was used to collect data with all participants. |
| 20 | Field notes | Were field notes made during or after interview or focus group | Yes, field notes were made during semi-structured interviews and focus group discussions |
| 21 | Duration | What was the duration of interview or focus group? | Individual interviews among different community groups lasted between 40 and 60 minutes, while semi-structured interviews with health professionals and community health workers lasted between 20 minutes and 40 minutes.  Focus group discussion with the general population lasted between 60 and 80 minutes. |
| 22 | Data saturation | Yes, data saturation was discussed | Yes, data saturation was discussed during data collection. |
| 23 | Transcript returned | Were transcript returned for participants for comments and or/comments | No. Transcriptions were not returned to each individual participant. But, meetings were held with some participants to discuss the preliminary findings of data analysis. |
|  | Domain 3: Analysis and findings | | |
|  | Data analysis | | |
| 24 | Number of data coders | How many data coders coded the data? | 3 |
| 25 | Description of the coding tree | Did authors provide the description of coding tree? | Yes. |
| 26 | Derivation of themes | Were themes identified in advance or derived from the data? | Some themes were identified in advance. However, the analysis used open coding, which enabled the identification of the others themes and subthemes emerging from the data. |
| 27 | Software | What software, if applicable, was used to manage the data? | NVivo 12 (QRS International Pty) |
| 28 | Participants checking | Did participants provide feedback on the findings? | Yes, through community meetings organized in the community to discuss the preliminary findings. |
|  | Reporting | | |
| 29 | Quotations presented | Where participants quotations presented to illustrate the themes/findings/Was each quotation identified? | Yes. But each quotation was labelled two general social characteristics. This method aimed at preserving the identity of the participants. |
| 30 | Data and finding consistence | Was there consistence between the data presented and the findings? | Yes. |
| 31 | Clarity of major themes | Were major themes clearly presented in the findings? | Yes. |
| 32 | Clarity of minor themes | Is there a description of diverse cases or discussion of minor themes? | Yes. |
