# Supplementary material for: Acceptability and perceived barriers to reactive focal mass drug administration in the context of a malaria elimination program in Magude district, Southern Mozambique: A qualitative study
Source: PLoS One. 2023 Mar 31;18(3):e0283160. doi: 10.1371/journal.pone.0283160 (PMC10065238; doi:10.1371/journal.pone.0283160)
Supplement: Attachment — Submitted filename: Response to Reviewers.docx [file pone.0283160.s008.docx]

**Subject: Submission of the revised manuscript [PONE-D-22-12696]**

Dear academic editor and reviewers,

Thank you for reviewing the manuscript “Acceptability and perceived barriers to reactive focal mass drug administration in the context of a malaria elimination program in Magude district, Southern Mozambique: A qualitative study”. The authors of this manuscript have read the current Instructions for Authors, and agreed to accept the recommedned format. The new manscript version reflects the recommeded format. All authors have also read and agreed upon the submitted version of this manuscript. We believe that the new manuscript will now be suitable for publication format in the PLOS ONE journal.

**To acdemic editor:**

1. Please ensure that your manuscript meets PLOS ONE's style requirements, including those for file naming. The PLOS ONE style templates can be found at
   <https://journals.plos.org/plosone/s/file?id=wjVg/PLOSOne_formatting_sample_main_body.pdf> and 
   <https://journals.plos.org/plosone/s/file?id=ba62/PLOSOne_formatting_sample_title_authors_affiliations.pdf>

**Answer**: We followed the recommeded format and we used the PLOS ONE tampletes to revised the manuscript. The new manuscript reflect the recommened format.

2. You indicated that you had ethical approval for your study. In your Methods section, please ensure you have also stated whether you obtained consent from parents or guardians of the minors included in the study or whether the research ethics committee or IRB specifically waived the need for their consent.

**Answer**: The research procol was approved by local and national IRB, namely CISM’s institutional ethics committee (CIBS-CISM) and the Mozambican Ministry of Health National Bioethics Committee, and a consent was obtained from parents and guardiens of the minor included in the study. In addition, an assent was obtained from the young adolecents that participated in the study. This information was now added in Methods section, particularly in etihcal consideration section of the new manuscript version. In addition, Table 1 was reformulated for better reading of the presented data.

3. **Data availability**

We agree and we support the policy of data availability, and we recognize the advantages of data availability. We have read PLOS ONE policy and we think that is very important to share the data publicly. However, the qualitative data used to develop this manuscript involve human discourses, and therefore, there is ethical and legal restrictions to sharing the data publicly. The ethical and legal restriction derive from the fact that the protocol and the informed consent and assent approved by the two ethical review boards referred that the data would only be available to the study team, and the protocol established that all information would be confidential. Thus, no participant of the study was informed that the data would be made publicly. Despite this restriction, the data of this study may be available to all researchers upon request to IRBs. In this regard, we would like to update our statement of data availability to as follows:

**Data Availability:** The data of this study were collected under individual-level informed consent and assent after a research protocol was reviewed and approved by CISM’s institutional ethics committee (CIBS-CISM) and the Mozambican Ministry of Health National Bioethics Committee. The informed consent signed by the participants stated that: “data will only be available to the study team”, and the protocol stablished that all information will be confidential, and no data from the data collection forms, nor from audio files will be accessible to anyone outside of CISM. Given this statement approved by the two IRBs, data from this study is available upon request to these institutional review boards: CISM’s institutional ethics committee ([sozinho.acacio@manhica.net](mailto:sozinho.acacio@manhica.net)) or the Mozambican Ministry of Health National Bioethics Committee ([jflschwalbach@gmail.com](mailto:jflschwalbach@gmail.com)) for researchers who meet the criteria for access to confidential data.

4. We note that you have indicated that data from this study are available upon request. PLOS only allows data to be available upon request if there are legal or ethical restrictions on sharing data publicly. For more information on unacceptable data access restrictions, please see <http://journals.plos.org/plosone/s/data-availability#loc-unacceptable-data-access-restrictions>.

**Answer**: The answer for this question was already provided in question 3.

5. "PLOS requires an ORCID iD for the corresponding author in Editorial Manager on papers submitted after December 6th, 2016. Please ensure that you have an ORCID iD and that it is validated in Editorial Manager.

**Answer**: The correspondent author already has an ORCID iD, which is **0000-0002-6871-1218.** I update this ORCID iD in Editorial Manager page, in Update my information, as recommened.

6.Please include captions for your Supporting Information files at the end of your manuscript, and update any in-text citations to match accordingly. Please see our Supporting Information guidelines for more information: <http://journals.plos.org/plosone/s/supporting-information>.

**Anwer**: The captions were included for the supporting information as recommended. The supported information include the intergiew guides used for data collection (in both Portguese and English) and Table COREQ. It does not include tables or data mentioned in the manuscript.
