# Supplementary material for: Acceptability and perceived barriers to reactive focal mass drug administration in the context of a malaria elimination program in Magude district, Southern Mozambique: A qualitative study
Source: PLoS One. 2023 Mar 31;18(3):e0283160. doi: 10.1371/journal.pone.0283160 (PMC10065238; doi:10.1371/journal.pone.0283160)
Supplement: Attachment — Submitted filename: Evaluation Report Submission for PONE-D-22-12696_R1 Manuscript.docx [file pone.0283160.s009.docx]

Manuscript Number: PONE-D-22-12696_R1

| General Comments: | This is an interesting and insightful study. However, the tables in the results sections were not properly presented. Few grammatical errors and spellings are seen. Hence, the authors need to revise the manuscript following the suggestions made. Then it is good to be published. Accept. |
| --- | --- |
| Abstract | - Line 38: Correct need with ‘needed’ |
| Introduction | - Line 59: Consisted of not Consisted (in) remove - Line 74-77: Rephrase or replace with ‘Although, there is significant reduction in cases of malaria in the district, elimination of Malaria…………………’ Hence, the WHO recommendation of …………………………surveillance, as an intervention …………………………………….. - Line 82: add after and ‘by the’ - Line 86: remove administrated replace with ‘administered’ and replace ‘of’ with ‘to’ - Line 93: incentivising?? Was an incentive given? If not, I suggest you use ‘encouraging’ - Line 98-100: Stop at interventions, then delete from ‘such as……..to general members of the community’. When deleted, in Magude district will end the sentence. |
| Methodology | Good.  Line 174: Reconfirm. Is it researched or reached?  Line 185: Remove ‘Moreover, replace with ‘Additionally’ |
| Results | Needs complete revision. Please adapt your tables with examples of PLOS ONE tables. Put full stops too at decimal places and not commas. % is written above and not all over the tables. |
| Discussion | Line 1109: Replace with perform malaria test**s on** children  Line 1111: test not testing  Line 1113: Husband’s not husband  Line 1114: about a positive not about positive |
| Others: |  |
| Decision | **Can be Published after these corrections.** |

Overall rating using the following: (1 = Excellent) (2 = Good) (3 = Fair) (4 = Poor)

| Originality: | 2 |
| --- | --- |
| Contribution to the Field: | 2 |
| Technical Quality: | 2 |
| Clarity of Presentation: | 2 |
| Depth of Research: | 2 |
