# Supplementary material for: Acceptability and perceived barriers to reactive focal mass drug administration in the context of a malaria elimination program in Magude district, Southern Mozambique: A qualitative study
Source: PLoS One. 2023 Mar 31;18(3):e0283160. doi: 10.1371/journal.pone.0283160 (PMC10065238; doi:10.1371/journal.pone.0283160)
Supplement: Attachment — Submitted filename: Response to Reviewers.docx [file pone.0283160.s011.docx]

## Subject: Submission of the revised manuscript [PONE-D-22-12696]

Dear editor and reviewers,

Thank you for reviewing our manuscript “Acceptability and perceived barriers to reative mass focal drug administraction in the context of malaria elimination program in Magude district. Southen Mozambique: a qualitative study”. The authors of this manuscript have read the comments and suggestions, and agreed to accept the recommedned conditions. All authors have also read and agreed upon the submitted version of the manuscript.

**To the Editor:**

In addition to the minor comments from the reviewers, I realized the authors have used over 30 pages to describe their results. It is important that the authors re-summarize these findings. I have also attached a marked-up revised copy for other comments.

Answer: The authors re-summarized the findings, and only the essential quotations were left. The new revised manuscript has 28 pages.

If you would like to make changes to your financial disclosure, please include your updated statement in your cover letter.

Answer: The authors added some data related to financial disclosure, but this did not change the statement at all. We have included the new text of financial disclosure in the cover letter.

**Answer to the Reviewer #1:**

- Typographic error in the manuscript:

Answer: all typographical errors identified were addressed. Moreover, the authors reviewed the whole manuscripts and corrected all possible grammatical errors.

**- Results**: Please adapt your tables with examples of PLOS ONE tables. Put full stops too at decimal places and not commas. % is written above and not all over the tables.

Answer: all tables were revised and formatted according to PLOS ONE tables, as presented in the new revised manuscript.

**Abstract**
1. "The reactive focal mass drug administration (rfMDA) was implemented July 2017 to January 2020". Authors should include in the abstract at what period between July 2017 to January 2020 was study conducted.

Answer: The authors included the period that the study was conducted in the abstract, which was between June and September 2017.

**Methods**
1. In Galatas et al. 2021 in "Community acceptability to antimalarial mass drug administrations in Magude district, Southern Mozambique: A mixed methods study", a population of 48,448 residents, and 10,965 households was provided for a study done during rounds of MDA between November 2015 to February 2017. In this study also conducted in 2017, authors present a population of 63,691inhabitants and 14,583 households.

Can authors give explanation to the difference in population number in studies done within same period?

**Answer**: Since the start of the project, the CISM conducted annual census in the district, and Galatas et al. (2021) reported the population of the local census of 2016. However, in the paper, we used the census of the National Institute of Statistic (INE) of 2017 rather than local census because the CISM did not conduct the 2017 local census. We therefore, decided to use the census of INE because we think it is updated compared to the 2016 local census.

2." The study was undertaken in September 2017 before the start of the reactive surveillance intervention and continued during the first two months after the start of the intervention".

a. The above contradicts the initial mention that rFMDA started in July 2017. If the people of Magude district did not benefit from intervention until after September 2017, authors should explain this in the Introduction to support what was mention in the Methods section. Otherwise, corrections should be made in the Methods (Study setting) section to agree with the earlier mention.

Answer: Indeed, there was an error in the text regarding the period of the study and the start of rfMDA intervention. The correct information is that the study was conducted between June and September 2017, while the rfMDA program started in September 2017. We have corrected this information in the revised manuscript.

b. In the Methods section (Study setting), authors should state the duration of study. That is, from September 2017 to when, 2018?

Answer: There was also an error in this section about the study period. The correct information is: the study was conducted between June and September 2017.

3. "...distributed in 5 Administrative Posts: Magude village, Motaze, Mahele,Panjane and Mapulanguene , and the study covered all these 5 Administrative Posts". In Table 1, some administrative units lacked participants bearing in mind in study purposive sampling was employed. Can authors give reasons why some units lacked particpants?

**Answer**: The study sample size did not cover all participants in all study sites due to unequal distribution of the study participants’ categories and several constraints to accessing the eligible participants. Health professionals and CHWs were not included in some study sites because they were not in all selected communities, and some community leaders were absent during the data collection. Additionally, it was not possible to include adolescents from Mahele and Mapulanguene because the study took place during the school season, and the eligible participants were at schools in different districts. Furthermore, members of the community from Mahele were not included in the sample of semi-structured interviews because they were unavailable due to their agricultural activities. In addition, the lack of accessibility at the selected study sites during the rainy season constrained the eligible participants' access. Moreover, the data collection strategy used also contributed to unequal distribution of the participants in the study setting. The study started by conducting focus group discussions (FGDs) in all study settings and later carrying semi-structured interview. While FGDs were conducted during late months of dry season, the semi-structured interviews were conducted during the start of rainy season, which constrained access to participants in some study settings (Administractive post of Motaze, Mahele and Mapulanguene). we have added an explanation of the sample distribution in the revised manuscript (page 10 of the revised manuscript with track changes).

4. Was the semi-structured interview questionnaire used pre-piloted?

Answer: Yes, all guides were pre-piloted as started in line 205, page 11 of the revised manuscript with track changes.
